# Combining ECM Hydrogels of Cardiac Bioactivity with Stem Cells of High Cardiomyogenic Potential for Myocardial Repair

**DOI:** 10.1155/2019/6708435

**Published:** 2019-10-23

**Authors:** Rui Bai, Lei Tian, Yi Li, Jiao Zhang, Yujie Wei, Zhigeng Jin, Zhiqiang Liu, Huiliang Liu

**Affiliations:** ^1^Department of Cardiology, The Third Medical Center of Chinese PLA General Hospital, 69 Yongding Road, Haidian District, Beijing 100039, China; ^2^Beijing Institute of Basic Medical Sciences, 27 Taiping Road, Haidian District, Beijing 100850, China

## Abstract

Tissue engineering exploring the combination of scaffolds and seeding cells was proposed as a promising strategy for myocardial repair. However, the therapeutic outcomes varied greatly due to different selection of scaffolds and seeding cells. Herein, the potential of combining bioactive extracellular matrix (ECM) hydrogels and high cardiomyogenic seeding cells was explored for myocardial repair *in vitro* and *in vivo*. Temperature-sensitive ECM hydrogels were prepared from decellularized rat hearts, and cardiomyogenic seeding cells were isolated from brown adipose (brown adipose-derived stem cells (BADSCs)). The *in vitro* studies demonstrated that ECM hydrogel significantly supported the proliferation and cardiomyogenic differentiation of BADSCs. Importantly, the function and maturation of BADSC-derived cardiomyocytes were also promoted as evidenced by Ca^2+^ transient's measurement and protein marker expression. After myocardial transplantation, the combination of BADSCs and ECM hydrogels significantly preserved cardiac function and chamber geometry compared with BADSCs or ECM hydrogels alone. Meanwhile, the ECM hydrogel also enhanced BADSC engraftment and myocardial regeneration *in vivo*. These results indicated that heart-derived ECM hydrogels exerted significant influence on the fate of cardiomyogenic cells toward benefiting myocardial repair, which may explain the enhanced stem cell therapy by the scaffold. Collectively, it indicated that the combination of ECM hydrogel and the cardiomyogenic cells may represent a promising strategy for cardiac tissue engineering.

## 1. Introduction

Ischemic cardiovascular disease (CVD) is one of the major causes of morbidity and mortality worldwide [1, 2]. Due to the limited regenerative potential of postnatal cardiomyocytes, an initial coronary artery blockage would result in progressive cardiomyocyte loss and eventually heart dysfunction or even heart failure [3, 4]. Consequently, exogenous regeneration with stem cells was proposed for myocardial repair and widely investigated [5, 6].

Transplanted stem cells may promote myocardial repair through a paracrine effect or regeneration. However, most studies observed rare regeneration previously, and thus, the major contributor for the improved heart function was ascribed to paracrine effects, which was usually of short term. Actually, due to the limited regeneration from exogenous cells, clinical practice benefited little from laboratory achievements in stem cell-based therapy of MI [7–9]. Two major factors may be responsible for the limited regeneration of transplanted stem cells: (1) the very low cardiomyogenic potential of seeding cells themselves [10, 11] and (2) the cardiomyogenic potentials and survival of seeding cells may be further impaired by a hostile microenvironment [9]. Therefore, it is vital to explore a type of seeding cells with high cardiomyogenic potentials and minimize the adverse influence of an engrafted microenvironment, so as to improve stem cell-based myocardial repair and further the clinical application in the future.

In recent years, the application of suitable scaffolds was considered one of effective strategies to improve stem cell survival against an adverse microenvironment. Among various scaffolds reported, natural extracellular matrix (ECM) derived from the decellularized organs was confirmed as a promising one for stem cell transplantation, because they could largely preserve not only the native tissue-specific composition and ultrastructure but also the resident cytokines [12, 13]. Independent studies have demonstrated that the scaffolds were efficient in regulating cell behaviors and improving the therapeutic outcome of seeding cells [14, 15], including for myocardial repair [16]. However, its potential in cardiac tissue engineering was far from thoroughly explored. As mentioned above, a seeding cell with great cardiomyogenic potential was also crucial for myocardial repair. Though kinds of cells have been investigated as seeding cells for MI, including mesenchymal stem cells (MSC), cardiac stem cells (CSC), and embryonic stem cells (ESC), most of them were either of low cardiomyogenic potential (such as MSC) or hardly available in a clinic (such as CSC and ESC). Previously, we have reported an efficient method for acquiring brown adipose-derived stem cells (BADSCs) with high cardiomyogenic potential from young rats instead of neonatal ones [17], making it possible for the seeding cells in therapeutic application. The great cardiomyogenic potential suggested that the cells would be promising in cardiac tissue engineering. However, the potential of the seeding cells for myocardial repair was largely unclarified.

In the present study, temperature-sensitive ECM hydrogels were prepared using decellularized heart matrix. The effects of the hydrogel on the proliferation, cardiac differentiation, and mutation were systemically evaluated *in vitro*. Then, the combination of BADSCs and temperature-sensitive ECM hydrogels was explored for cardiac regeneration and repair in MI models.

## 2. Materials and Methods

### 2.1. Preparation of the Decellularized Heart ECM

Adult Sprague-Dawley (SD, about 250 g) rats were anesthetized by intraperitoneal injection of pentobarbital sodium (30 mg/kg bodyweight), and then, the hearts were fully exposed. 15 mL heparinized PBS (10 IU/mL) was injected through the inferior vena cava to prevent blood coagulation; the hearts were dissected from the chest with an intact aortic arch structure and emerged in the heparinized PBS solution. A 16G blunt end needle was then inserted into the aorta of the heart for retrograde perfusion with 1% SDS for 6 hours, followed by 1% Triton X-100 for 0.5 h at a speed of 3 mL/min in a closed liquid circulation system. After that, PBS solution was used for heart perfusion to remove the reliquus detergent. PBS solution was replaced at least 3 times each day, and at least 4-day perfusion was performed.

### 2.2. Characterization of the Decellularized Intact Heart ECM

After fixation with 2.5% glutaraldehyde for 24 h, the decellularized heart was dehydrated with gradient ethanol solutions and dried under vacuum condition. The ECM was then treated with spray-gold and observed with a scanning electron microscope (Hitachi S-3400N).

For the histological examination, the fresh heart and decellularized heart were fixed with 4% paraformaldehyde. 3-5 *μ*m paraffin-embedded sections were prepared for hematoxylin and eosin (H&E) staining and immunostaining assay. For immunostaining, sections were incubated with the primary antibodies overnight at 4°C and then incubated with FITC-labeled secondary antibodies. DAPI (Solarbio, Beijing) was then used for nuclear staining. The images were recorded with a fluorescent microscope (Olympus, Japan). The primary antibodies collagen I (1 : 150, Boster, Wuhan, China), collagen III (1 : 150), fibronectin (1 : 150), laminin (1 : 150), *α*-actinin (1 : 200, Sigma), and cTnT (1 : 200; Sigma) were used.

### 2.3. Preparation of the ECM Hydrogel

Decellularized ECM hydrogel was prepared according to a previous repot [16]. Briefly, the left ventricular wall section of the decellularized intact heart ECM was digested with pepsin-HCl solutions (1 mg/mL in 0.1 M HCl for 48 h under constant stirring at a pepsin : matrix ratio of 1: 10). After that, pH values were adjusted to 7.4 with sodium hydroxide (NaOH). The ECM gel was then diluted to a final concentration of 6 mg/mL with 1× PBS and kept at -20°C before using. The solubilized myocardial matrix gel was also characterized using SDS-PAGE.

### 2.4. Cell Seeding on Col or ECM-Coated Glass Substrates

The interscapular brown adipose tissues was derived from male SD rats (70 ± 10 g). Isolation and cultivation of BADSCs were performed according to a previous report [17] with slight modification. Briefly, brown adipose tissues were extensively washed with sterile phosphate-buffered saline (PBS) and cut into small size (<1 mm^3^). The tissues were digested for 40-60 min with 0.1% trypsin+0.1 mg/mL collagenase IV+0.1 mg/mL dispase II in a serum-free medium. The digestion was terminated by adding serum-containing medium, and the solution was centrifuged at 600g/min to collect the cells. The cells were suspended in *α*MEM supplemented with 10% fetal bovine serum (FBS). For routine cultivation, the cells were seeded into culture plates in *α*MEM supplemented with 10% FBS and incubated under 37°C, 5% CO_2_ condition. To investigate the influence of substrate on cell behaviors, glass slides of 1 cm × 1 cm size were coated with Col (collagen I) or ECM as previously reported [18]. Briefly, the cleaned glass slides were fully covered with Col I or ECM solution (6 mg/mL, *w*/*v*) to form a thin layer. After that, the substrate was dried by stoving at 37°C or lyophilization. Before cell seeding, the coated slides were preincubated with *α*MEM for at least 6 h. 5 × 10^4^ of BADSCs were then seeded on the slides and cultured in *α*MEM medium containing 10% FBS, 100 U/mL penicillin, and 100 *μ*g/mL streptomycin.

### 2.5. Live/Dead Assay

After incubation for 1 and 3 days, the LIVE/DEAD® Cell Vitality Assay Kit (L34951, Thermo Fisher Scientific, USA) was used to test cell viability according to the manufacturer's instructions. The stained cells were observed using an inverted fluorescent microscope (Olympus IX71, Japan), and the images were obtained from 3 random fields of each sample (*n* = 5) to calculate the live and dead cell numbers.

### 2.6. Alamar Blue Assays and BrdU Labeling

After culture for 1, 3, 7, and 14 days, the cells were then incubated with 10% Alamar Blue reagent (in culture medium) for12 h [19] at 37°C. The cell viability was then assessed by measuring the Alamar Blue fluorescence (*n* = 5). For BrdU labeling, the cells were incubated with 5-bromo-2′deoxyuridine (BrdU; Sigma) after 3 days of culture. The positive cells for BrdU were detected by anti-BrdU antibodies and observed with an inverted fluorescent microscope (Olympus IX71, Japan).

### 2.7. Cardiac Differentiation of BADSCs

In order to investigate the effects of ECM on cardiac differentiation of BADSCs, immunofluorescent staining, Western blotting, and spontaneous Ca^2+^ transients were performed. For immunofluorescent staining, BADSCs were seeded on glass substrates and cultured for 7 days. Then, the cells were fixed with 4% paraformaldehyde. After permeabilization with 0.3% Triton, the cells were incubated with primary antibodies against cTnT and connexin-43 (CX43) at 4°C overnight. After washing with PBS, the cells were incubated with Rhodamine Red-X- or AF488-conjugated donkey anti-mouse or rabbit secondary antibodies (Boster, China). Nuclei were stained with DAPI. The images were captured using an Olympus fluorescent microscope.

For Western blotting, the BADSCs were lysed, and then, total protein was extracted and quantified using a BCA protein assay kit (Pierce, Thermo Fisher Scientific, USA). 30 *μ*g of total proteins was loaded onto 15% SDS-containing polyacrylamide gel for electrophoresis (SDS-PAGE). The separated protein bands were then transferred onto polyvinylidene difluoride (PVDF) membrane (Immobilon-P, Millipore) and blocked by 5% nonfat skimmed milk for 60 min at room temperature. The samples were incubated with primary antibodies overnight at 4°C, followed by incubating with HRP-conjugated secondary goat anti-rabbit antibody (1 : 8000, Beyotime, Beijing) for 90 min at room temperature. Finally, band was detected with chemiluminescence. Primary antibodies against cTnT (1 : 2000), CX43 (1 : 2000), and GAPDH (1 : 8000) were purchased from CST (Cell Signaling Technology, USA).

For spontaneous Ca^2+^ transient measurement, BADSCs were incubated with 4 *μ*M fluo-4 AM (Molecular Probes, Eugene, OR) for 30 min at 37°C and then washed with serum-free medium for three times. The Ca^2+^ transients were recorded by a LSM-710 laser scanning confocal microscope (Carl Zeiss, Inc. Germany) operating in the frame (X-Y) imaging mode. Fluorescent imaging of intracellular Ca^2+^ dynamics was acquired at a rate of 3 ms per scan.

### 2.8. Myocardial Infarction and Cell Transplantation

All animal experiments were approved by the Institutional Animal Care and Use Committee (IACUC) of the Beijing Institute of Basic Medical Science (Beijing, China). The myocardial infarction was prepared by ligating the left anterior descending coronary artery as reported previously [6]. The animals were randomly divided into 4 groups: (1) PBS-treated group, (2) PBS+BADSC- (or BADSC-) treated group, (3) ECM-treated group, and (4) ECM+BADSC-treated group. For implantation treatment, the 100 *μ*L of PBS or ECM with or without 5 × 10^6^ BADSCs (DiI labeled) was injected into the infarct bed through the 30-gauge needle.

### 2.9. Functional Evaluation and Histological Examination

4 weeks after surgery, rats were anesthetized and echocardiography (14.0 MHz, Sequoia 512; Acuson) was performed for cardiac function evaluation. The left ventricular end-diastolic diameter (LVEDD) and left ventricular end-systolic diameter (LVESD) were measured. The left ventricular shortening fraction (LVFS %) and left ventricular ejection fraction (LVEF %) were calculated as previously reported [6].

The animals were euthanized with overdose of pentobarbital sodium after functional evaluation. The hearts were harvested and fixed. Paraffin-embedded sections were prepared with standard protocols. 3-5 *μ*m sections were prepared and stained with Masson's Trichrome staining solution. The infarct size and left ventricle wall thickness were quantified as previously reported on Masson's trichrome images [6]. The immunohistochemical staining against VWF (1 : 200, Boster, Wuhan, China) and *α*-SMA (1 : 200, Boster, Wuhan, China) was performed to analyze the vessel densities in an infarct zone using the same protocol in Section 2.2.

### 2.10. Statistical Analysis

All data were expressed as the mean ± standard deviation. The data from the two groups were analyzed by Student's *t*-test, and the data from more than two groups were analyzed by one-way or two-way ANOVA with Tukey's post hoc test. A value of *p* < 0.05 was considered statistically significant. Statistical analysis were performed with OriginPro 8.0.

## 3. Results

### 3.1. Decellularization and Gelation of Myocardial Matrix

**Figure 1:**
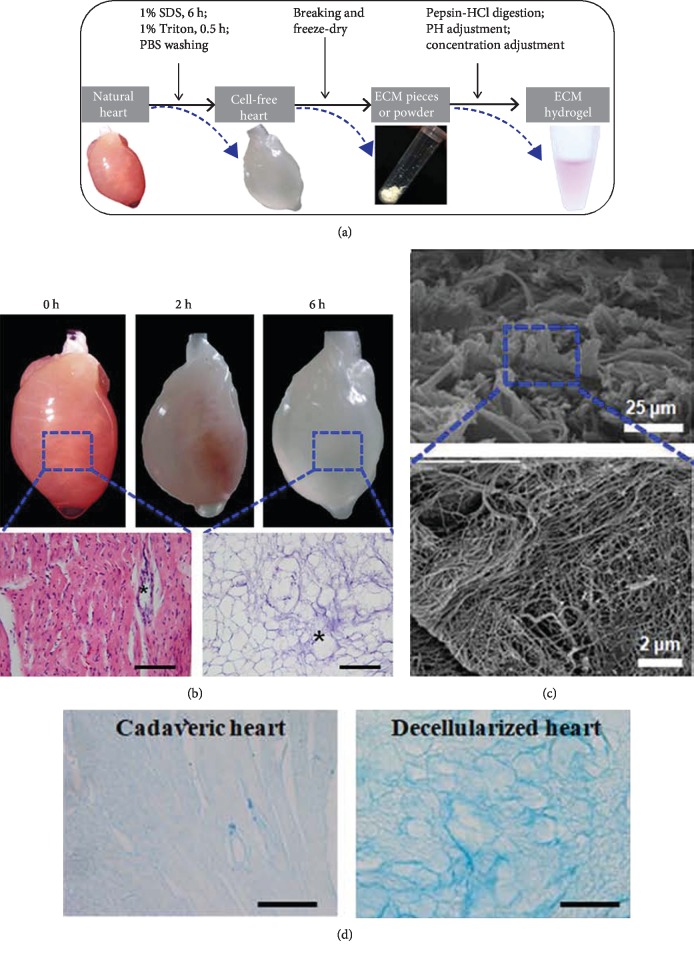
Preparation of decellularized heart ECM. (a) The schematic procedures of preparing decellularized heart hydrogels. (b) Observation of decellularized heart and H&E staining. (c) Scanning electronic observation of decellularized ECM. (d) Alcian Blue staining of natural and decellularized hearts.

Figure 1 demonstrates the procedure of preparing the myocardial ECM and hydrogels. After 6.5-hour perfusion, the heart became fully translucent and resulted in a fully decellularized heart with an intact three-dimensional structure (Figure 1(b)). H&E staining demonstrated that the cells were completely removed and intact vascular basal membranes were preserved. SEM observation of decellularized heart showed the presence of myocardial matrix composition and no cardiac cells (Figure 1(c)). Alcian Blue staining of natural and decellularized hearts confirmed the good preservation of the glycosaminoglycans (Figure 1(d)).

**Figure 2:**
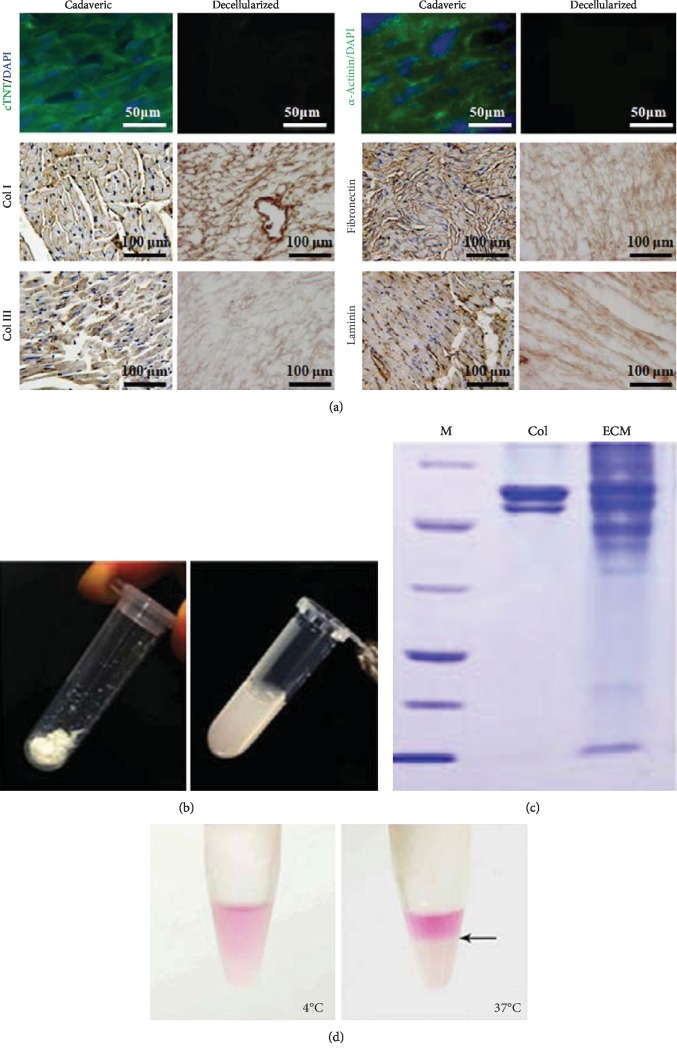
Characterization of decellularized heart matrix and preparation of ECM gel. (a) Immunostaining against cardiac markers, cTNT, and *α*-actinin, as well as extracellular matix components, 8 Col I, fibronectin, Col III, and laminin. (b) Preparation of ECM gel. (c) SDS-PAGE analyzing the components of ECM hydrogel. (d) Thermosensitive feature of the ECM hydrogel.

Immunostaining on decellularized heart sections detected no nuclei or contractile elements (cTnT and *α*-actinin) but detected the extracellular components, including collagen types I and III, fibronectin, and laminin (Figure 2(a)). The decellularized ECM was then treated with pepsin to prepare the ECM gel (Figures 1(a) and 2(b)). Gel electrophoresis of the ECM gel demonstrated its more complicated components than Col I (Figure 2(c)). Much more bands of different molecular weights were present in decellularized matrix, indicating the preservation of multiple kinds of ECM components. What is more, the dissolved ECM was dissolved at 4°C, while it gelatinized via self-assembly at 37°C (Figure 2(d)), demonstrating a thermosensitive feature.

### 3.2. The Influence of ECM Substrates on BADSC Survival and Proliferation

**Figure 3:**
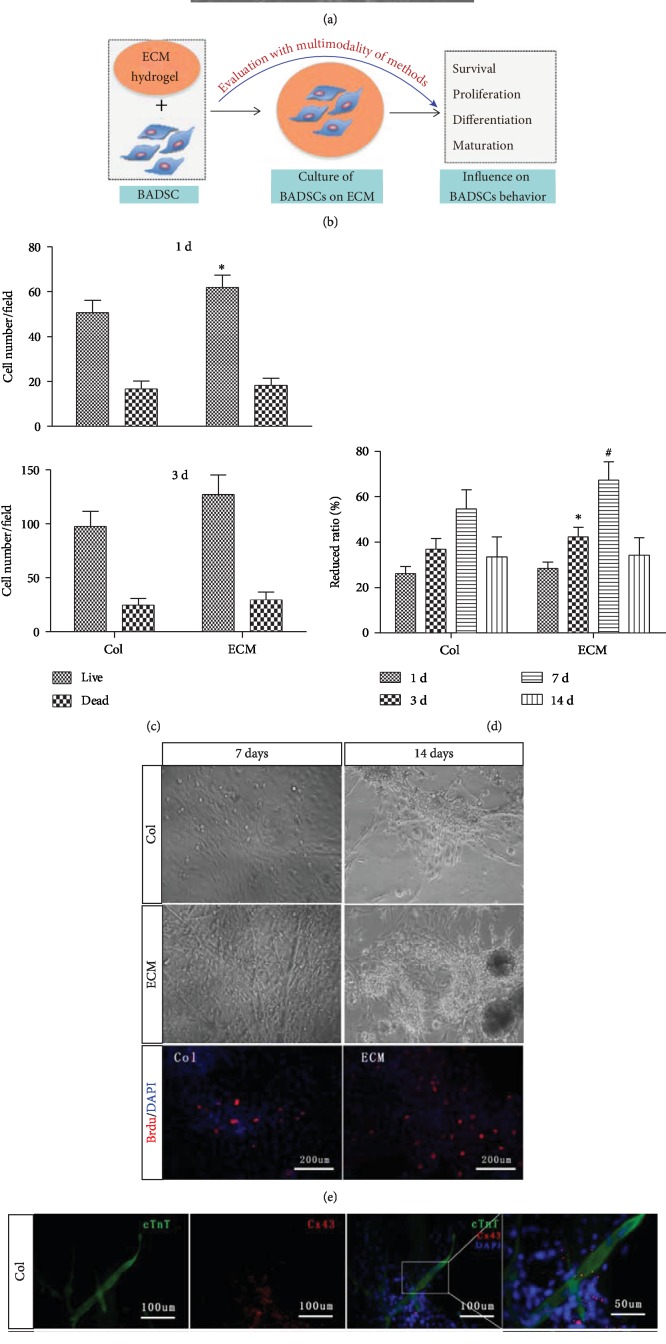
Evaluation of ECM on the BADSC proliferation and differentiation. (b) The schematic summary of evaluating the influence of ECM hydrogel on BADSC behaviors. (c) Live and dead cell number counting after 1 day and 3 days of culture. (d) Alamar Blue assays demonstrated that both ECM and Col I could promote the proliferation of BADSCs. (e) Morphological observation of BADSCs with time grown on Col I or ECM hydrogel, as well as BrdU labeling of BADSCs at 3 days after seeding. (f) Immunofluorescent staining of cTnT (green) and CX43 (red) expression in BADSCs. ^∗^*p* < 0.05 and ^#^*p* < 0.01 for “ECM vs. Col” at the same time points.

The morphology of BADSCs under routine cultivation is shown in Figure 3(a).  Figure 3(b) illustrates the experimental procedures to investigate the effects of ECM on the BADSC behavior. BADSCs were seeded on ECM-coated substrates. Col I-coated substrates were used as control. Cell count at 1 day and 3 days showed that ECM substrates significantly enhanced the adhesion and survival of BADSCs as shown in Figure 3(c).

Alamar Blue assays demonstrated that both ECM and Col I could promote the proliferation of BADSCs within 7-day cultivation. However, in comparison, cell viabilities at 3 and 7 days were significantly higher in ECM-coated substrates than in Col I-coated ones (Figure 3(d)). After 14-day cultivation, contact inhibition of BADSCs was observed on both ECM- and Col I-coated substrates (Figure 3(e)). BrdU labeling further confirmed the enhanced proliferation of BADSCs on ECM substrates compared with those on Col I (Figure 3(e)).

### 3.3. The Influence of ECM Substrates on BADSC Differentiation

**Figure 4:**
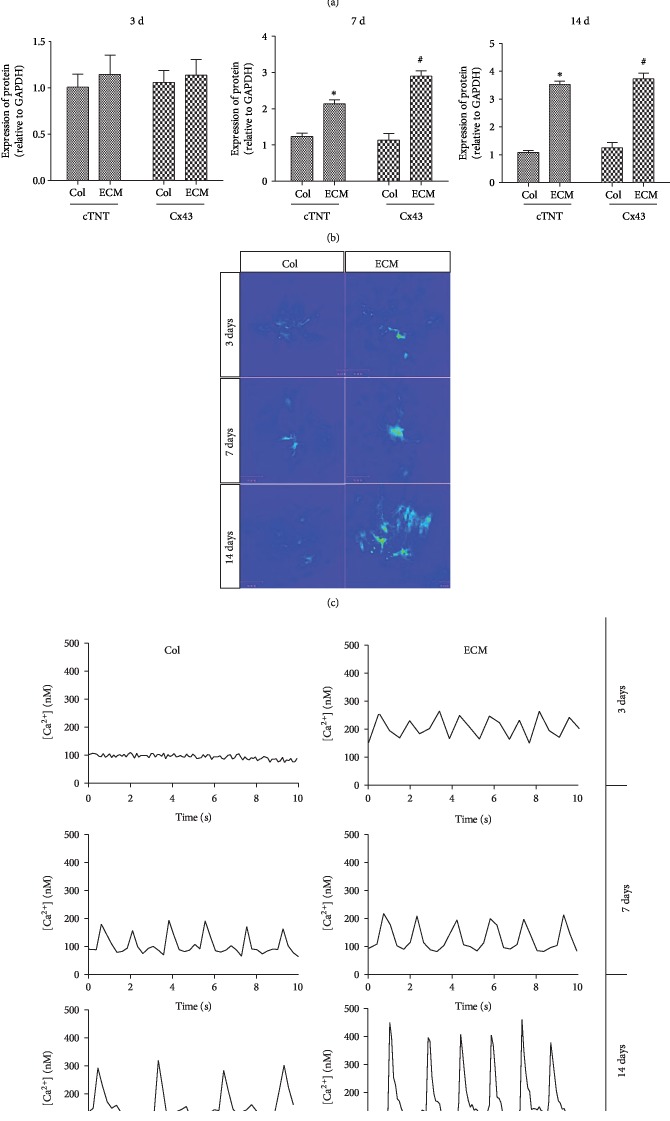
Cardiac differentiation and maturation of BADSCs on ECM-coated substrates. (a) Western blotting detecting the expression of cardiac markers cTnT and CX43. (b) Quantitative analysis of protein expression in BADSCs. (c, d) Ca^2+^ transient imaging of BADSCs on Col I and ECM-coated substrates. ^∗^*p* < 0.05 and ^#^*p* < 0.01 for “ECM vs. Col” at the same time points.

To evaluate the effect of ECM on the differentiation of BADSCs, cardiac-specific proteins and spontaneous Ca^2+^ transients were tested. Immunofluorescent staining showed a higher expression of intercalated disc-related proteins (cTnT and CX43) in BADSCs growing on ECM substrates (Figure 3(f)) than those growing on Col I substrates. The expression of cTnT and CX43 was further confirmed by Western blotting (Figures 4(a) and 4(b)).

The cardiac activities were further evaluated by spontaneous calcium transients. As shown in Figures 4(c) and 4(d), calcium transients of differentiated BADSCs grown on ECM substrates displayed more regular and higher levels of Ca^2+^ amplitudes than those growing on Col I substrates, indicating that ECM substrate promoted the maturation of BADSC-differentiated cardiomyocytes.

### 3.4. The Influence of ECM Gel on the Therapeutic Performance of BADSCs

**Figure 5:**
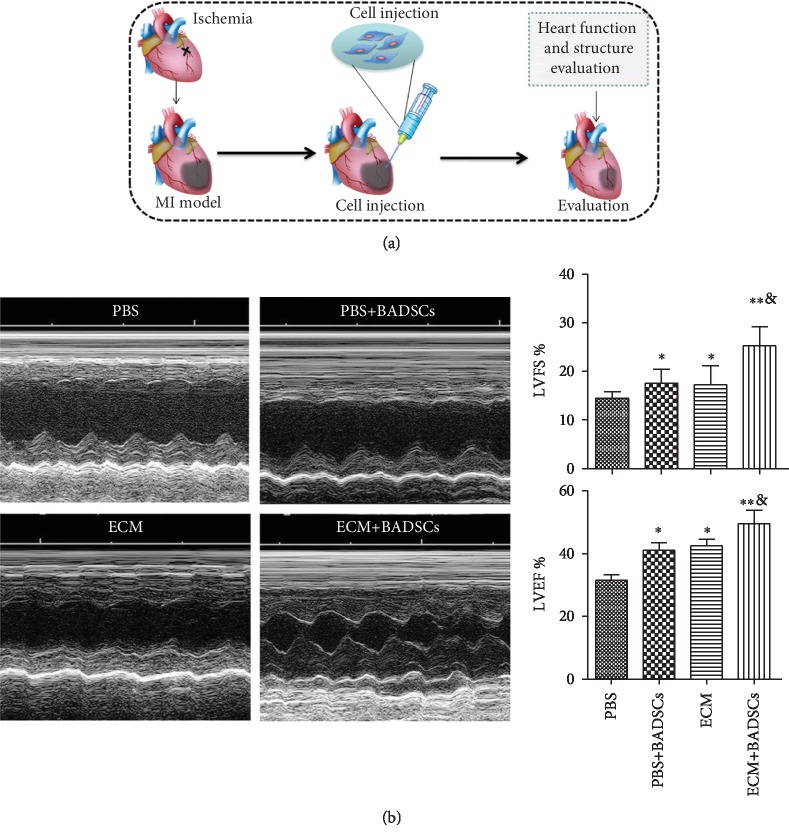
Cell transplantation and functional evaluation. (a) The schematic summary of MI preparation and cell transplantation. (b) Heart function evaluation by echocardiogram and quantitative analysis of functional parameters, LVFS and LVEF. ^∗^*p* < 0.05 compared with control group; ^∗^*p* < 0.01 compared with control group; ^&^^∗^*p* < 0.05 compared with the BADSC and ECM alone groups.

ECM gel was used as injectable scaffolds to deliver BADSCs for treating MI of SD rats.  Figure 5(a) illustrates a simple procedure of BADSC transplantation by using ECM hydrogel as carriers. At week 4, functional evaluation by echocardiography demonstrated that ECM and BADSCs alone could slightly attenuate myocardial injury after MI compared with PBS control (*p* < 0.05) (Figure 5(b)). However, heart function was significantly improved in rats treated by the combination of ECM and BADSCs compared with the control group (*p* < 0.01), as well as the ECM and BADSC-treated groups; (*p* < 0.05). The results suggested that ECM gels as scaffolds significantly improved the therapeutic performance of BADSCs for MI.

### 3.5. Histological Examination

**Figure 6:**
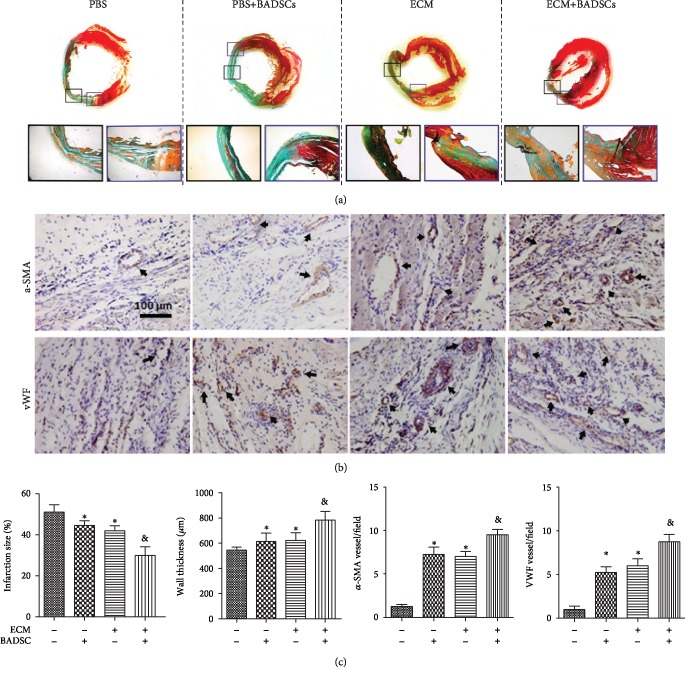
Histological examination of injured heart. (a) Masson's trichrome staining. (b) The immunohistochemical staining of vWF and *α*-SMA for microvessel density analysis in the myocardial infarction sites. Black arrows indicated positively stained vessels. (c) Quantitative analysis for infarct size, wall thickening, and vascular densities in infacted areas. ^∗^*p* < 0.05 and ^∗∗^*p* < 0.01 for “BADSCs or ECM vs. Col” and ^&^*p* < 0.01 for “BADSCs or ECM vs. BADSCs+ECM”.

To further evaluate the influence of ECM hydrogel on the therapeutic performance of BADSCs for MI, histological examination was performed at 4 weeks after implantation. Masson's trichrome staining showed that the infarct size was decreased and the wall thickness was increased in the ECM and BADSC groups compared with the PBS group, while the beneficial effects were further enhanced in the ECM+BADSC group (Figure 6(a)). Immunohistochemical staining against vWF and *α*-SMA detected a higher level of vascularization in the ECM+BADSC-treated group than the other groups (Figure 6(b)). Quantification and statistical analysis showed that histological parameters, including infarct size, wall thickness, and vascular density, were all significantly superior in the ECM+BADSC group to other groups (Figure 6(c), *p* < 0.01 compared with other groups).

**Figure 7:**
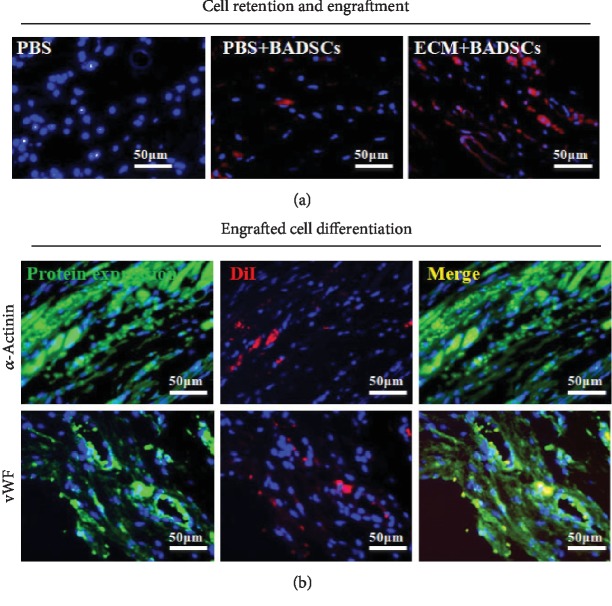
*In vivo* survival and cardiac differentiation of BADSCs. (a) Histological examination of DiI-labeled BADSCs 4 weeks after implantation (b) and immunofluorescent staining against *α*-actinin and vWF in the ECM+BADSC group.

Further, the engraftment of transplanted BADSCs in the hearts was identified by DiI fluorescence (Figure 7(a)). It could be observed that more DiI-positive cells appeared in the ECM+BADSC group than in the BADSC alone group, indicating the carrier hydrogel enhanced the retention and engraftment of transplanted cells. Then, the colocation of DiI and cardiovascular markers was detected so as to determine the differentiation of BADSCs in ischemic hearts. No colocation of DiI and cardiovascular markers was detected in the BADSC alone group (data not shown), while very rare cells in the ECM+BADSC group were observed (Figure 7(b)). These results suggested that ECM gel may also promote the cardiovascular differentiation of BADSCs in an infracted myocardium, and meanwhile, direct cardiovascular differentiation could contribute little to improve heart function as the differentiated cells were so few.

## 4. Discussion

The application of biomaterials as scaffold has been proved beneficial for MSC-based therapy in different disease models [20–22]. According to disease types, selection of scaffolds and seeding cells was crucial to achieve a satisfied therapeutic outcome, e.g., a scaffold suitable to an ischemic microenvironment and a seeding cell with potent cardiomyogenic potential were important for MI. In the present study, heart-derived ECM hydrogel was selected as a scaffold to facilitate BADSC transplantation in MI for the first time. The novel findings of the study included the fact that (1) decellularized heart ECM hydrogel could positively regulate the behaviors of BADSCs. This would be instructive to develop novel scaffolds for BADSC regulation and myocardial regeneration. (2) The combination of ECM hydrogels and BADSCs significantly promoted myocardial repair compared with BADSCs alone, suggesting an effective way to treat myocardial infarction with BADSCs, promising seeding cells with potent cardiomyogenic potential.

Transplantation of stem cells has been proposed as a novel treatment for myocardial infarction for several years, and encouraging results have been achieved [23, 24]. However, the controversy existed all the time about the stem cell alone for the repair of injured myocardium and sustained functional improvement [9, 25]. The application of scaffolds was commonly considered a promising strategy to enhance stem cell therapy [26–28]. However, the related research varied greatly in the past years due to the different choices of scaffolds [29, 30]. Ideally, a good scaffold should be bioactive and provide a growth environment close to a natural extracellular matrix [31–33]. Apparently, it was hard for a synthetic scaffold to meet this requirement considering the complexity of an *in vivo* environment. Natural organ-derived ECM or modified ECM may be the optimal option. Compared with other biomimetic scaffolds as previously reported [34–37], decellularized ECM may create a more favorable niche for stem cells as they preserved lots of bioactive proteins or growth factors [12, 13] and thus mimicked tissue-specific natural extracellular microenvironments better. In fact, the bioactivities and benefits of decellularized ECM as scaffolds have been confirmed by independent groups [38–40]. Previously, though ECM-based scaffolds were explored in cardiac tissue engineering by different groups, no one has investigated its combination with high cardiomyogenic cells for myocardial repair. We supposed that an efficient strategy for cardiac tissue engineering should also include a good seeding cell, not only an ideal scaffold. Therefore, our purpose in the study was to explore the therapeutic efficiency of combining such ECM hydrogel with BADSCs, a seeding cell of high cardiomyogenic potential.

In the study, the decellularized ECM from the hearts preserved an intact 3D native heart structure and various extracellular matrix components, including collagen, laminin, fibronectin, and GAGs. These results suggested that the material should possess a good bioactivity of natural heart ECM, which may explain its positive regulation on BADSCs as observed in the study. To enhance the applicability of the decellularized ECM, it was solubilized via pepsin digestion and prepared as injectable hydrogel that would be compliant for different injury sites. In addition, the injectable myocardial ECM hydrogel was proved to be temperature-responsive, which may be more favorable for cell retention in the myocardium because the hydrogel would gelate rapidly after injection *in vivo*. Previous studies indicated that the decellularized heart ECM could sustain the function of seeding cells, such as the cell survival and proliferation, and it can also modulate the cardiac differentiation of embryonic stem cells as well as lineage-restricted progenitor cells [16, 41]. With glass slides coated with decellularized myocardial ECM, the beneficial effects of the scaffold on BADSCs were investigated and consistent results were achieved as previously reported that the ECM-coated substrates can promote the early attachment (24 hours) and proliferation of BADSCs. Interestingly, ECM also promoted the cardiac differentiation and maturation of BADSCs compared with Col I as proved by high expression levels of cTnT and CX43 proteins and better performance of spontaneous Ca^2+^ transients.

After myocardial transplantation in rat models, the improved therapeutic outcomes of BADSCs by ECM gel were observed as multifold, including increased survival of transplanted cells and LV wall thickness, decreased infarct size, and enhanced LV function. In addition, the revascularization of injured hearts was also improved. The results indicated that the ECM gel may also promote the secretion of angiogenic factors from BADSCs, such as VEGF, which may be another contributor to the enhanced therapeutic outcome of BADSCs. However, the potential influence of the decellularized myocardial ECM on the paracrine capability and other functions were not directly revealed in the present study, which deserved an in-depth investigation in the future.

In conclusion, our results indicated that the decellularized heart ECM could preserve intact chamber geometry of native heart and most extracellular matrix components. Thus, the resulted hydrogels from ECM possessed a good bioactivity and positively regulated the behaviors of BADSCs in favor of myocardial repair, including cell survival, proliferation, and cardiac regeneration. The myocardial ECM hydrogels we prepared in the study may represent a promising scaffold in cardiac tissue engineering. In addition, the cardiomyogenic BADSCs together with the ECM hydrogel would be a promising strategy for MI therapy.

## Data Availability

The data used to support the findings of this study are included within the article.
